# A novel approach to evaluating the UK childhood immunisation schedule: estimating the effective coverage vector across the entire vaccine programme

**DOI:** 10.1186/s12879-015-1299-8

**Published:** 2015-12-29

**Authors:** Sonya Crowe, Martin Utley, Guy Walker, Jasmina Panovska-Griffiths, Peter Grove, Christina Pagel

**Affiliations:** Clinical Operational Research Unit, University College London, 4 Taviton Street, London, WC1H 0BT UK; Department of Health, Area 330, Wellington House, 133-155 Waterloo Road, London, SE1 8UG UK

**Keywords:** Childhood immunisation programme, Modelling framework, Effective coverage

## Abstract

**Background:**

The availability of new vaccines can prompt policy makers to consider changes to the routine childhood immunisation programme in the UK. Alterations to one aspect of the schedule may have implications for other areas of the programme (e.g. adding more injections could reduce uptake of vaccines featuring later in the schedule). Colleagues at the Department of Health (DH) in the UK therefore wanted to know whether assessing the impact across the entire programme of a proposed change to the UK schedule could lead to different decisions than those made on the current case-by-case basis. This work is a first step towards addressing this question.

**Methods:**

A novel framework for estimating the effective coverage against all of the diseases within a vaccination programme was developed. The framework was applied to the current (August 2015) UK childhood immunisation programme, plausible extensions to it in the foreseeable future (introducing vaccination against Meningitis B and/or Hepatitis B) and a “what-if” scenario regarding a Hepatitis B vaccine scare that was developed in close collaboration with DH.

**Results:**

Our applications of the framework demonstrate that a programme-view of hypothetical changes to the schedule is important. For example, we show how introducing Hepatitis B vaccination could negatively impact aspects of the current programme by reducing uptake of vaccines featuring later in the schedule, and illustrate that the potential benefits of introducing any new vaccine are susceptible to behaviour changes affecting uptake (e.g. a vaccine scare). We show how it may be useful to consider the potential benefits and scheduling needs of all vaccinations on the horizon of interest rather than those of an individual vaccine in isolation, e.g. how introducing Meningitis B vaccination could saturate the early (2-month) visit, thereby potentially restricting scheduling options for Hepatitis B immunisation should it be introduced to the programme in the future.

**Conclusions:**

Our results demonstrate the potential benefit of considering the programme-wide impact of changes to an immunisation schedule, and our framework is an important step in the development of a means for systematically doing so.

**Electronic supplementary material:**

The online version of this article (doi:10.1186/s12879-015-1299-8) contains supplementary material, which is available to authorized users.

## Background

The UK’s routine childhood immunisation programme aims to protect children against a number of preventable infectious diseases over the first 5 years of life. At the time of this work (August 2015), the current programme protects against 11 different diseases and is scheduled as shown in Table 1. The schedule has evolved over time and is expected to further evolve in response to changing circumstances, such as the availability of new vaccines.

**Table 1:**
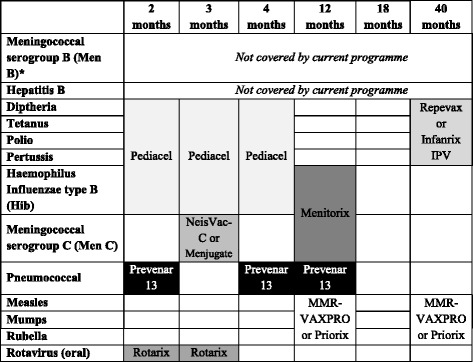
The current UK childhood vaccination schedule

The current childhood vaccination schedule in the UK by age of the child, showing the diseases covered and the vaccines that can be used (brand names given). ^a^Note that the introduction of Meningococcal serogroup B (Men B) has been agreed but has not yet started

Decisions to change the immunisation programme are currently made on a case-by-case basis, informed primarily by cost-effectiveness analysis specific to the vaccine change being considered [1–5]. Typical changes might involve introducing a new vaccine to protect against a disease not previously covered, or changing the vaccine product used to protect against a disease already in the programme.

However, alterations to one aspect of the schedule, whether to the timing of existing vaccinations or the introduction of a new vaccine product, may have implications for other areas of the programme. For example, there are potential mechanisms whereby adding more injections to the schedule could reduce the uptake of vaccines that feature later in the schedule [6–12]. For instance, the likelihood that a parent brings their child to a particular visit may depend on the number of visits they have already attended (visit fatigue) and/or whether any of these visits involved a “bad experience”, as well as the perceived importance and safety of each vaccine scheduled for that visit. This introduces the scope for changes to the programme having less overall benefit than anticipated - or even causing net harm.

Given this, it is important to consider whether assessing the overall programme-level impact of a proposed change to the schedule could lead to different decisions than those made on a case-by-case basis. However, at present, decision-makers do not have a systematic way to do this. For example, there is no framework for exploring the impact that a particular change to the immunisation schedule might have on the coverage achieved for other diseases that occur later in the programme and thus the potential consequences on the overall burden of vaccine preventable disease on the population. Authors from the UK Department of Health (DH) (GW and PG) asked the other authors to explore collaboratively the feasibility of developing such a framework that could be used for future UK immunisation planning.

Within the context of the US immunisation schedule, previous work has considered the optimal formulation of combination vaccines using integer programming, incorporating schedule-relevant constraints and capturing the associated costs [13–17]. Jacobson et al. [13] developed a web-based tool that enables decision-makers to use economic factors beyond vaccine purchase price to design childhood vaccine formularies within a given context, including the value of existing and new combination vaccines. In contrast to this previous work, we do not consider costs in this paper and instead focus our study on developing a framework to estimate how changes to the vaccine programme could impact the effective coverage against each disease in order to inform decision making, given the current schedule. With the intent of illustrating how a programme-view of vaccine procurement could be beneficial, we apply the framework to the current UK childhood immunisation programme (August 2015) and explore a selection of feasible future changes to the schedule. The parameter space and set of constraints we consider is thus smaller than in Jacobson et al.’s work. The development and application of our framework is an important first step towards evaluating the programme-level impact of changes in a given childhood immunisation programme.

## Methods

**Fig. 1:**
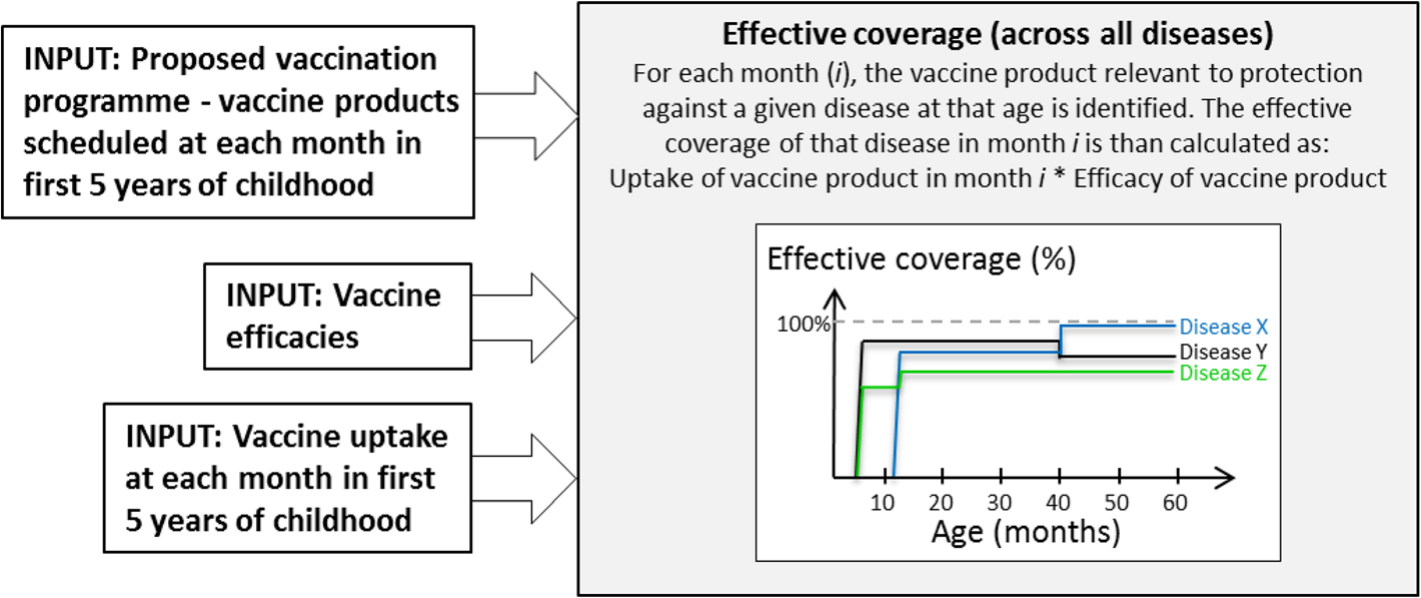
A schematic of the modelling framework developed in this paper. Using inputs on the effectiveness of vaccines and vaccine uptake, the framework estimates the age-dependent effective coverage against each of the diseases, calculated as the product of vaccine uptake and vaccine efficacy

A modelling framework for evaluating vaccine schedules was developed (Fig. 1) in order to estimate the effective coverage that would be achieved against each disease included in a given vaccination programme (section Developing a modelling framework to estimate the effective coverage against all diseases within a schedule). To demonstrate the potential use of our approach, we parametrised (section Inputs for the modelling framework) and applied (section Application of the framework to different vaccine scenarios) the framework to the current childhood vaccination programme in the UK (August 2015) and plausible future extensions to this vaccination programme. Our modelling approach does not use a dataset or database but rather draws on parameters estimated from the literature, expert opinion or vaccine manufacturer information (as referenced in the text).

### Developing a modelling framework to estimate the effective coverage against all diseases within a schedule

**Fig. 2:**
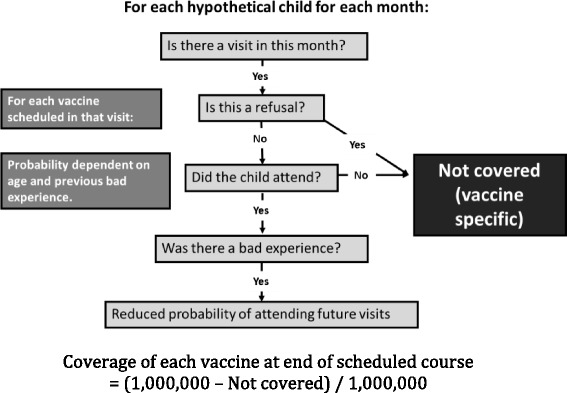
Schematic of the simulation used to estimate vaccine uptake. This shows the logical flow of the simulation model developed to implement Part I of the framework in this work. The simulation is used to estimate the age-dependent uptake of each vaccine in the immunisation programme being considered

One way in which the introduction of a new vaccine could impact on the entire schedule is through influencing the uptake of other vaccines. Therefore we developed a simple method to estimate the effective coverage that would be achieved for each disease included in a given vaccination programme among a cohort of children at each month over the first five years of life. Effective coverage against a given disease was calculated as the product of the uptake of the vaccine and the vaccine efficacy. We used a simple simulation model to estimate uptake, the key features of which are illustrated in Fig. 2. Within the simulation framework, parameters regarding the immunisation schedule, efficacies of the relevant vaccines and assumptions around vaccine uptake (section Inputs for the modelling framework) are used to calculate an estimated vector of effective coverage across all diseases for each of the first 60 months of life. Parameter estimates were based on the literature and expert advice, and tuned such that the coverage estimated from the simulation is consistent with recent national data on uptake from the Cover of Vaccination Evaluated Rapidly (COVER) dataset [18]. We note that exact fits to COVER data would not be expected since COVER is a cross-sectional dataset and the simulation is a longitudinal cohort model. COVER data was only used to check that our assumptions regarding age-dependent attendance and bad experiences led to plausible estimates of uptake at 1 year, 2 years and 5 years. All parameter estimates were agreed with the Department of Health (DH) in the UK and can be changed by the user (see below for details).

#### Assumptions regarding vaccine uptake

Within the model, we assume that every child is eligible for each vaccine (i.e. contra-indications are ignored). Several studies have reported that parent attitudes towards vaccination vary by disease and they are less likely to vaccinate their children against diseases that are not perceived as a threat, whilst a small percentage of parents may ‘opt out’ of vaccination entirely (1.1 % [12]). In the model we therefore incorporated a percentage of ‘non-participants’ for each of the vaccines, and split the vaccines into three groups defined by their relative likelihood of refusal (Table 2). We also set a baseline age-dependent probability of ‘participants’ attending a visit each month (Table 3), accounting for the fact that children are less likely to be taken for routine GP visit as they get older (in line with evidence that parents find later visits harder to organise and that there is lower uptake for booster doses [9, 10]). Stockwell et al. [11] showed in a US study that a previous bad experience during vaccination increased the likelihood of later under immunisation and Tickner et al. [10] reported a negative impact of bad experiences on future visits. We wanted to incorporate scope for these behavioural effects to impact coverage within the model. We therefore estimated the probability of a bad experience for any single vaccination to be 1 % and assume that a bad experience reduces the probability of further visits by 20 % (Table 3). We note that different assumptions around the chance of a bad experience and the impact on future visits could have been made for the same overall effective coverage. Our aim was to choose plausible estimates consistent with observed uptake data that could be changed should future research provide better evidence. Finally, primary and booster courses were treated separately in the model with uptake switching to that of the booster dose after its completion. Partial uptake of a primary course was not considered and we do not allow a missed visit to be completed later within an extra visit.

**Table 2:** Estimated percentage of parents refusing a vaccine for each vaccine considered

Vaccines	% children whose parents refuse vaccine
Pediacel (primary and booster); Repevax; Infanrix Hexa (primary and booster); Infanrix Penta (primary and booster); Bexsero	0.5 %
Meningitec (primary and booster); Menitorix (primary and booster); M-M-RVAXPRO (courses 1 and 2); HBVaxPro; Rotarix	1.1 %
Prevenar 13 (courses 1 and 2)	1.5 %

Model inputs for the probability of a participant parent taking their child for a scheduled GP visit at each age, with and without having a previous bad experience

**Table 3:** Model inputs for the probability of attendance

Age (months)	Probability of attendance (no bad experience)	Probability of attendance (after bad experience)
0-12	98.5 %	79 %
12-24	94.5 %	76 %
24-60	93.5 %	75 %

Model inputs for the probability of a participant parent taking their child for a scheduled GP visit at each age, with and without having a previous bad experience

### Inputs for the modelling framework

#### Diseases covered and vaccine products available

Within our framework we first identified the set of diseases/causative agents that are either already covered as part of the UK childhood immunisation programme (Diphtheria, Pertussis, Tetanus, Polio, Neisseria Meningitides Group C, Haemophilus influenzae B, Measles, Mumps, Rubella, Pneumococcal disease), or that were considered by DH authors to be possible candidates for inclusion in the programme within the foreseeable future (Hepatitis B and Meningitides Group B). We note that at the time of conducting the work, the recent decision to introduce Meningitis B vaccination to the programme had not been made and a procurement contract was agreed (as of end March 2015) just before submission of this work. Hepatitis A and Varicella were not included on the basis that, on cost-effectiveness grounds, they were less likely to be included in the schedule in the medium term. We compiled a list of available vaccine products relevant to these diseases [19–21].

#### The current immunisation schedule (GP visits)

At the time of writing (August 2015), the current childhood immunisation programme in the UK comprises five scheduled General Practitioner (GP) visits, at 2, 3, 4, 12–13 months and 40–60 months old with immunisation against 11 diseases (see Table 1).

#### Establishing plausible alternative immunisation schedules (GP visits)

Assuming it nonviable to withdraw vaccination against any disease currently targeted, we firstly manually explored alternative schedules for delivering vaccination against the diseases targeted by the current programme. We then extended this by allowing the possible addition of vaccination against Hepatitis B, Meningitis B, or both Hepatitis B and Meningitis B. In each case we constructed feasible vaccine schedules and attendant product combinations under five simplifying assumptions. Firstly, we assumed that additional vaccinations in the programme would, where possible, fit into existing scheduled GP visits, which would remain in the scheduled visits of the current programme (unless removed entirely), and where this was not possible we minimised the number of additional visits. Secondly, we assumed that no more than 3 injections would be given in any one GP visit within infancy (up to 1 year of age) but that after 1 year, a child can receive 4 injections in any one visit [DH, private communication]. We note that the Joint Committee on Vaccination and Immunisation (JCVI) have recommended the introduction of the Men B vaccine at months 2, 4 and 12 [22, 23], which would require four injections in the visit at month 12, so we explicitly allow for this option. Thirdly, we assumed that a disease cannot be vaccinated against more than once within the same visit (i.e. a disease can only be vaccinated by one product at any one time). Fourthly, we ignored vaccination against diseases not explicitly included in the programme (e.g. as a by-product of a combination vaccine). Finally, if two or more vaccine products provide protection against the same diseases under the same schedule then they were considered within the same option. We used the Electronic Medicines Compendium [20] to determine possible vaccine schedules where needed.

#### Vaccine efficacies

The efficacies of all the relevant vaccine products are given in Table 4, although we note that the user is able to adjust these inputs in the implementation of our simulation. This information originates from three main sources: the website for The European Medicines Agency (EMA) [19], the Electronic Medicines Compendium (EMC) [20], and the 2011 Green Book [21]. The sources reporting the efficacies were not in a consistent format and were given for different time points during the vaccination schedule. We used the efficacy corresponding to the percentage of children protected for lowest effective antibody effect as soon as possible following the final dose of the primary course of the vaccine. If a range of efficacies were given or different efficacies given for different disease serotypes, we used the average efficacy. The recommended dosing schedule and known interactions of the vaccine products with other vaccines were considered and we note that there are no significant interactions between any vaccines using our plausible immunisation schedules. Within the model, we assume this to be the efficacy at the point of completing the vaccination course (with no efficacy before completion) and that this efficacy does not wane over the time period considered (5 years). For diseases that require a booster, the primary vaccine efficacy is replaced with the efficacy of the booster once it has been administered. Some additional vaccine-dependent assumptions were also incorporated in the model (see footnotes in Table 4).

**Table 4:** Vaccines considered, the diseases they vaccinate against and the efficacies used in the modelling

Vaccine	Diseases vaccinated (efficacy used for modelling)	Source
Menjugate or NeisVac Primary (<1 year old)	Men C (0.994)	EMC
Menjugate or NeisVac Booster (>1 year old)	Men C (1)	EMC
Menitorix Primary (<1 year old)	Men C (0.993), Hib (1)	EMC
Menitorix Booster (>1 year old)	Men C (0.98 different primary, 1 otherwise), Hib (1)	EMC
MMR VAXPRO/Priorix after one dose (>1 year old)	Measles (0.90), Mumps (0.64), Rubella (0.99)	Green Book
MMR VAXPRO/Prioirix after two doses^a^ (>1 year old)	Measles (0.99), Mumps (0.87), Rubella (0.999)	Green Book
Pediacel (<1 year old)	Tetanus (1), Polio (1), Diphtheria (0.992), Pertussis (0.987), Hib (0.91)	EMC
Pediacel (>1 year old)	Tetanus (1), Polio (1), Diphtheria (0.991), Pertussis (0.967), Hib (0.991)	EMC
Prevenar 13 (both doses)	Pneumococcal (0.948)	EMC
Repevax or Infanrix IPV (>3 years old)	Tetanus (1), Polio (1), Diphtheria (1), Pertussis (0.995)	EMC
Rotarix (<1 year old)	Rotavirus (0.918)	EMC
HBVaxPRO (both doses)	Hep B (0.96)	EMC
Infanrix Hexa (<1 year old)	Tetanus (1), Polio (1), Diphtheria (1), Pertussis (1), Hib (0.964), Hep B (0.995)	European medicines agency
Infanrix Hexa (>1 year old)	Tetanus (0.999), Polio (0.999), Diphtheria (0.999), Pertussis (0.999), Hib (0.997), Hep B (0.984)	European medicines agency
Bexsero^b^	Men B (0.836)	EMC

^a^Efficacy after two doses takes into account greater likelihood of successful immune response after two doses. ^b^Efficacy for Bexsero is calculated as the product of the efficacy against the strains covered (0.95) and the proportion of Men B strains covered (0.88)

Vaccine-dependent modelling assumptions:

For the conjugate vaccines (PCV, Men B and Men C), a child who received only the booster at 12 months is assumed to be fully covered

For the DTaP/Hib vaccines, a child needs to have received both a primary course and a booster dose to be fully covered after the booster dose within the model (the primary course needs to be fully completed for the booster vaccination to offer protection)

For the MMR vaccine, a single dose is assumed to provide immunity but 10 % of children (randomly selected) don’t respond. A second dose gives these children another opportunity to be covered: this is not a booster as such, but a way of reducing the proportion of children who don’t respond. Within the model, we estimated the effective coverage for measles, mumps and rubella according to the number of children in the cohort who received none, one or two MMR vaccinations

For the Men B and Men C vaccines, efficacy was assumed to be zero after the age of ten (due to waning)

For the pneumococcal vaccine, we assumed efficacy was zero after the age of 15

### Application of the framework to different vaccine scenarios

To demonstrate how the framework could be used, we applied it to the current UK childhood vaccination schedule and each of the plausible schedules that had been established in order to compare the vectors of effective coverage. We then developed some illustrative examples, each involving the introduction of Hep B vaccination, to demonstrate how the programme-view of vaccine procurement could potentially lead to different decisions than the case-by-case approach. In Example 1, we introduce vaccination against Hep B to the current schedule using HBVaxPro, resulting in an extra GP visit at 10 months. In Example 2 , vaccination against Hep B is introduced to the current schedule by replacing the DtaP primary course (Pediacel) and booster (Repevax or Infanrix IPV) with Infranix Hexa and switching the Men C booster from Menitorix to NeisVac (or Menjugate Kit). In Example 3 we used the framework to examine a ‘what-if’ scenario of interest to policy makers around the possible impact of a negative media reporting that might impact on parents’ willingness to partake in aspects of the programme. This is a scenario of particular interest following the impact of the MMR scare in the UK, which reduced the coverage for MMR vaccines from 92 % in 1996 to 80 % in 2003 [24, 25]. This Hep B scenario is also relevant given the public concern and relatively low uptake of the flu vaccine in the UK [24] and reported parental concern and low uptake of the Hepatitis B vaccine in France [26, 27]. In this hypothetical scenario, we assumed that there was negative media reporting surrounding the introduction of the Infanrix Hexa vaccine for Hepatitis B that increased parental refusal of this vaccine (see for instance these 2009 UK media articles worrying about such an introduction [28, 29]). To model this, we use Option 5 (Table 6) but assume a higher proportion (5 % rather than 0.5 %) of parents refusing to attend for visits where Infanrix Hexa is administered. In addition to its impact on the coverage of all diseases targeted by the combination vaccine this would also affect the Men C, Rotavirus, Pneumococcal and MMR vaccinations. Our intention was to illustrate how strategies for targeting efforts to improve the vaccination programme could be informed by scenario analysis such as this, alongside considerations of feasibility and cost.

## Results

### Current and plausible future vaccination schedules

When exploring scheduling options for vaccinating against the 11 diseases that are currently covered by the immunisation programme in the UK (August 2015), we found only one alternative combination of vaccines to the current set that did not violate our constraints. This alternative involves an earlier booster vaccination for Diphtheria, Tetanus, Polio, and Pertussis that includes a (later) booster for Hib, switching to a single vaccine booster for Men C and an earlier MMR booster (see Table 5 in comparison with Table 1).

**Table 5:**
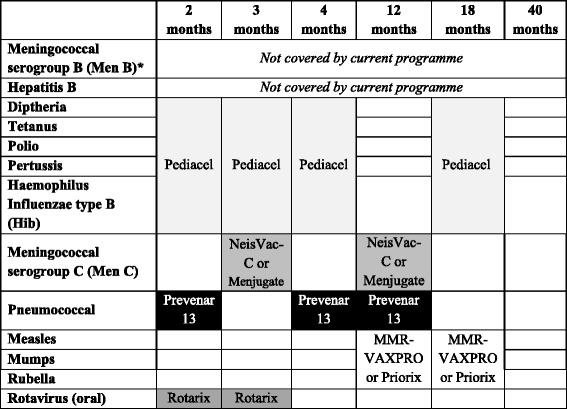
A plausible alternative vaccination schedule for the current UK childhood immunisation programme

An alternative to the current UK childhood vaccination schedule by age of the child, showing the diseases covered and the vaccines that can be used (brand names given). ^a^Note that the introduction of Meningococcal serogroup B (Men B) has been agreed but has not yet started

We also found that relatively few additional options become available with the introduction of further vaccines, allowing for explicit evaluation of all options. Specifically, our analysis shows that only three are plausible when Hep B is included, two when Men B is included and only one when both are included. The plausible options are set out in Table 6 and the scheduling of GP visits associated with each of these is presented in Additional file 1. When extending the current programme to include Hep B and/or Men B some interesting examples of potential trade-offs arise that are set out in the illustrative examples in Effective coverage. We also note that saturating a GP visit with the maximum number of injectable vaccines reduces flexibility for later adding the immunisation programme without additional visits. In particular, the planned introduction of Men B immunisation will fill up the infant schedule (given the reluctance to administer more than three injections in a single GP visit during infancy) such that future additions to this part of the schedule could be problematic.

**Table 6:** Vaccination options for the current UK childhood immunisation programme and plausible extensions to it in the foreseeable future

Diseases targeted	Option number	Vaccine products	Number of visits
Current programme: Diphtheria, Pertussis, Tetanus, Polio, Neisseria Men C, Hib, Pneumococcal disease, Measles, Mumps, Rubella and Rotavirus	Option 1	Pediacel; Repevax (or Infanrix IPV); NeisVac (or Menjugate Kit); Menitorix; Prevenar 13; MMR-VAXPRO (or Priorix); Rotarix	5
	Option 2	Pediacel; NeisVac (or Menjugate Kit); Prevenar 13; MMR-VAXPRO (or Priorix); Rotarix	5
As in current programme + Hepatitis B	Option 3	Pediacel; Repevax (or Infanrix IPV); NeisVac (or Menjugate Kit); Menitorix; Prevenar 13; MMR-VAXPRO (or Priorix); Rotarix; HBVaxPro	6
	Option 4	Pediacel; NeisVac (or Menjugate Kit); Prevenar 13; MMR-VAXPRO (or Priorix); Rotarix; HBVaxPro	6
	Option 5	Infanrix Hexa; NeisVac (or Menjugate Kit); Prevenar 13; MMR-VAXPRO (or Priorix); Rotarix	5
As in current programme + Men B	Option 6	Pediacel; Repevax (or Infanrix IPV); NeisVac (or Menjugate Kit); Menitorix; Prevenar 13; MMR-VAXPRO (or Priorix); Rotarix; Bexsero	5
	Option 7	Pediacel; NeisVac (or Menjugate Kit); Prevenar 13; MMR-VAXPRO (or Priorix); Rotarix; Bexsero	5
As in current programme + Hepatitis B + Men B	Option 8	Infanrix Hexa; NeisVac (or Menjugate Kit); Prevenar 13; MMR-VAXPRO (or Priorix); Rotarix; Bexsero	5

Four scenarios were considered: the current immunisation programme; adding Hepatitis B vaccination to the current programme; adding Meningitis B vaccination to the current programme; and adding both Hepatitis B and Meningitis B vaccination to the current programme. For each scenario we present the feasible combinations of vaccine products that could target the vaccine preventable diseases, each of which represents a plausible schedule option. Overall this gives rise to the eight possible options listed: the vaccination schedules for each of these options are contained in Additional file 1

### Effective coverage

**Fig. 3:**
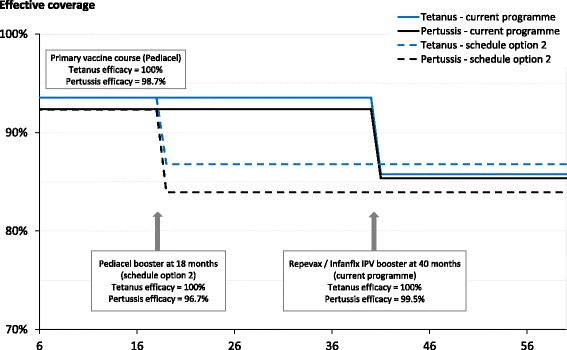
Estimated age-dependent effective coverage for Tetanus and Pertussis. The effective coverage of population aged 0 to 60 months for Tetanus and Pertussis estimated using the framework, given the current immunisation programme (solid lines) and Option 2 (an alternative programme for currently covered diseases, dashed lines)

Figure 3 shows the effective coverage over time for Pertussis and Tetanus, both for the current programme and Option 2 (the alternative feasible schedule for the current set of diseases). For pertussis, the effective coverage is always lower in schedule Option 2 than in the current programme because, despite the earlier booster in option 2 resulting in higher uptake of the booster, the vaccine efficacy of the Pediacel booster (Option 2) is quite a bit lower than for Repevax/Infanrix IPV. In contrast, for tetanus the 5 year effective coverage is higher for Option 2 than for the current schedule due to the earlier booster increasing uptake and the equal efficacy of the vaccine products in both cases. However, note that Option 2 is worse than the current schedule for the 22 months between the timings for the boosters for the two options since the primary course has a higher uptake than either booster and, within our framework, we assume there is no waning between the primary course and the booster. Although, if making decisions on timings, waning would need to be considered, this illustrates why it could be important to consider the time dependence of the effective coverage, particularly if the prevalence of the disease being vaccinated against has a strong age dependence.

**Fig. 4:**
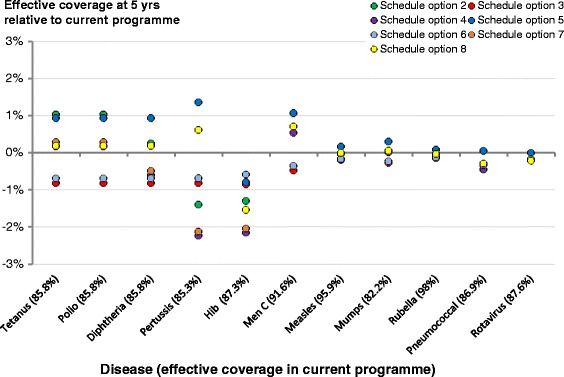
Relative effective coverage vector for Options 2–8. The framework was used to estimate the age-dependent effective coverage against each of the diseases within a given schedule. We present here the effective coverage against each disease at 5 years for Options 2–8, relative to the current vaccination regime. We have included the effective coverage under the current vaccination programme in the labels of the diseases

**Fig. 5:**
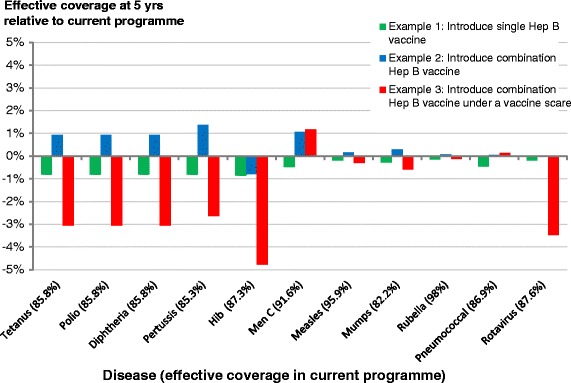
Relative effective coverage vector for illustrative examples. The effective coverage against each disease at 5 years relative to the current vaccination regime is shown for Examples 1–3. We have included the effective coverage under the current vaccination programme in the labels of the diseases

Figures 4 and 5 show the estimated effective coverage vector at 5 years relative to the current programme for Options 2–8 and the three illustrative examples respectively. From Fig. 4, we can see that both Option 7 (adding Men B as a single vaccine) and Option 8 (adding Hep B as a single vaccine) reduce the effective coverage of pertussis and Hib because they increase the chances of a bad experience and the possibility of non-attendance, thereby reducing coverage. The benefits gained from vaccinating against either disease would thus need to be weighed against this loss of coverage elsewhere.

From Fig. 5 we see that introducing Hep B by replacing the DtaP/IPV/Hib primary course and booster with Infranix Hexa and switching the Men C booster (Example 2) has benefits over and above the impact of Hep B by improving Pertussis, Hib, Men C protection (higher efficacy for all three), as well as MMR protection (higher coverage). This is a marked contrast to the generally worse effective coverage seen across the programme from Example 1 (Option 7, discussed above). Finally, through Example 3, where we compare the impact of introducing a combination vaccine to the current schedule with and without a vaccine scare, Fig. 5 illustrates how the potential benefits of introducing a new vaccine are susceptible to behaviour changes that affect uptake and so its introduction could potentially result in an overall worsening of the programme instead of an improvement.

## Discussion

We have developed a novel framework for estimating the effective coverage that would be achieved against each disease included in a given childhood immunisation schedule and applied this within the context of the UK vaccination programme.

Firstly, we showed in our analysis that, under certain assumed constraints, there is only one plausible alternative vaccine schedule to the current one, an interesting finding that had not been explicitly recognised by colleagues at DH prior to this work.

Secondly, when we applied the framework to plausible extensions of the current (August 2015) immunisation schedule in the foreseeable future (introducing vaccination against Men B and/or Hep B) and an additional scenario exploring the impact of negative media coverage of a new vaccine, we illustrated how it might be useful to evaluate the programme-wide impact of a particular change to an immunisation schedule in contrast to the case-by-case approach used at present. For instance, introducing a single Hepatitis B vaccination is shown in one of our examples (Example 1) to impact negatively on many parts of the existing programme because it reduces the overall uptake of vaccines featuring later in the schedule (by increasing visit fatigue and the chances of a bad experience), but introducing Hep B as part of a combination vaccination benefits the rest of the programme since the combination vaccine also has higher efficacy against already covered diseases. Interestingly, however, negative media coverage surrounding the introduction of Hepatitis B, could potentially result in an overall *worsening* of the programme benefit of introducing Hepatitis B to the current schedule even in the most beneficial schedule option (Example 3). In this illustrative example, a higher than normal proportion of parents refuse to attend for visits where the Infanrix Hexa vaccine is administered, which affects the uptake of the Meningitis C, Rotavirus, Pneumococcal and MMR vaccinations as well as Hepatitis B and the other diseases covered by the combination vaccine, with knock-on negative impact on programme benefit as a whole.

Within the context of the enormous progress over the last 60 years in protecting children against diseases such as Polio and Diphtheria, which are now virtually eliminated in the UK, the current immunisation programme arguably has greatest room for improvement in better protecting against: Pertussis (due to the high incidence [21] and relatively low efficacy of primary vaccine); Pneumococcal (for which there are many complications and some long term sequelae [30]) and; Rotavirus (which affects a lot of small children [2, 31]). Of the plausible extensions to the current programme, the introduction of Meningitis B vaccine may be particularly beneficial since it is serious disease that kills 1 in 10 people affected and leaves a further third with long lasting effects, some as serious as amputations, brain damage and hearing loss [1, 32]. Indeed, since conducting this work, the decision has been made to introduce Meningitis B vaccination into the childhood immunisation programme [23] and, as of March 2015, the UK government have negotiated a procurement contract to enable vaccination to start later in 2015 or early in 2016 [33] (its routine use is not yet included in the Meningococcal chapter of the Government’s Green Book [34]). This decision was based largely on considerations of its cost effectiveness and uncertainty around estimates of cost-effectiveness [22]. Within this context, our work provides a timely illustration of why it might also be useful to take a programme-view that considers the potential benefits and scheduling needs of all vaccinations on the horizon of interest rather than those of an individual vaccine in isolation. For instance, introducing Meningitis B immunisation into the current vaccination schedule would require an additional injection in the early (2 month) GP visit. Within our framework, this saturates that 2 month visit with the maximum number of injectable vaccines, which reduces flexibility for later introductions of other vaccines to the immunisation programme. For example, if immunisation against Hepatitis B was subsequently introduced to the programme (in addition to Meningitis B), this would only be achievable by switching certain other vaccine products (see schedule Option 8) or introducing another GP visit to the schedule. On the other hand, adding **both** Men B and Hep B to the schedule potentially provides **greater** overall gain to the immunisation programme (even excluding any benefit from preventing Hep B) than adding **only** Men B because the combination vaccine that includes Hep B offers better protection for Pertussis. This demonstrates how our framework can usefully augment existing cost-effectiveness analyses and why it may be important to take a more strategic view of procurement decisions.

This work was conducted in direct response to a specific request from DH to examine the potential usefulness of a programme-view in evaluating changes to the immunisation schedule. Thus one of the strengths of the work is that we collaborated with the DH to ensure that our work was informed by the nature of the potential decisions faced in this area. Aspects of the model development, parameterisation and analysis therefore focused on features of the current UK immunisation programme. However, we note that our framework is flexible in terms of input parameters and constraints and can therefore readily be applied to any vaccination programme and can easily accommodate possible alterations to incorporate future vaccination programme changes.

This work has successfully illustrated why it may be important to take a more strategic view of procurement decisions. We note, however, that a programme-wide evaluation of a schedule cannot include the level of detail incorporated in individual vaccine cost benefit analyses such as those currently performed when considering, for example, whether to introduce a particular vaccine. We therefore stress that this framework is not proposed as a replacement to cost-effectiveness analyses, but rather to sit alongside them in informing the difficult decisions that need to be made regarding vaccine procurement and options for improving the vaccination programme. We believe that the strategic view demonstrated using our framework offers a complementary approach to the current case-by-case one. However, whilst informative, the effective coverage vector does not provide an easy means for comparison across different diseases within a schedule or for the evaluation and comparison of different schedules at a programme level since it does not take into account disease burden (i.e. weighting the importance of effective coverage by severity and prevalence of the disease). Thus, the framework presented here represents an important starting point of what could be a larger-scale definitive framework able to inform national immunisation policy. For example, a natural extension of the framework would be to develop a platform for quantifying disease burden from the vector of effective coverage by incorporating disease-specific epidemiological models and, where necessary, developing models to project the number of disease cases under different vaccine scenarios.

## Conclusions

We have developed a novel framework for estimating the vector of effective coverage across all of the diseases in a given vaccination programme and applied it within the UK context. This is an important step towards providing decision-makers with a means for systematically exploring the programme-wide impact of a particular change to an immunisation schedule and assessing scope for further reducing the burden of vaccine preventable disease across a programme. Our analyses using the framework highlight illustrative circumstances in which taking such a programme view may be beneficial.

## Additional file

Additional file 1:
**The eight plausible immunisation schedules (GP visits).** (DOCX 54.9 kb)
