# Supplementary material for: A novel approach to evaluating the UK childhood immunisation schedule: estimating the effective coverage vector across the entire vaccine programme
Source: BMC Infect Dis. 2015 Dec 29;15:585. doi: 10.1186/s12879-015-1299-8 (PMC4696176; doi:10.1186/s12879-015-1299-8)
Supplement: Additional file 1: — The eight plausible immunisation schedules (GP visits). (DOCX 54.9 kb) [file 12879_2015_1299_MOESM1_ESM.docx]

**Additional File 1: The eight plausible alternative immunisation schedules (GP visits)**

The scheduling of GP visits associated with each of the 8 options for the childhood vaccination programme considered is presented below.

Option 1: Current schedule

Option 2: Modified current schedule

Option 3: Current schedule + Hep B

Option 4: Modified current schedule + Hep B

Option 5: Alternative current schedule incl. Hep B

Option 6: Current schedule + Men B

Option 7: Modified current schedule + Men B

Option 8: Alternative current schedule incl. Hep B and Men B
